# Elucidation of the molecular envenomation strategy of the cone snail *Conus geographus* through transcriptome sequencing of its venom duct

**DOI:** 10.1186/1471-2164-13-284

**Published:** 2012-06-28

**Authors:** Hao Hu, Pradip K Bandyopadhyay, Baldomero M Olivera, Mark Yandell

**Affiliations:** 1 Eccles institute of Human Genetics, University of Utah, and School of Medicine, Salt Lake City, UT, 84112, USA; 2 Department of Biology, University of Utah, Salt Lake City, UT, 84112, USA

**Keywords:** *Conus geographus*, Conotoxins, RNA-seq, Venom duct compartmentalization

## Abstract

**Background:**

The fish-hunting cone snail, *Conus geographus*, is the deadliest snail on earth. In the absence of medical intervention, 70% of human stinging cases are fatal. Although, its venom is known to consist of a cocktail of small peptides targeting different ion-channels and receptors, the bulk of its venom constituents, their sites of manufacture, relative abundances and how they function collectively in envenomation has remained unknown.

**Results:**

We have used transcriptome sequencing to systematically elucidate the contents the *C. geographus* venom duct, dividing it into four segments in order to investigate each segment’s mRNA contents. Three different types of calcium channel (each targeted by unrelated, entirely distinct venom peptides) and at least two different nicotinic receptors appear to be targeted by the venom. Moreover, the most highly expressed venom component is not paralytic, but causes sensory disorientation and is expressed in a different segment of the venom duct from venoms believed to cause sensory disruption. We have also identified several new toxins of interest for pharmaceutical and neuroscience research.

**Conclusions:**

*Conus geographus* is believed to prey on fish hiding in reef crevices at night. Our data suggest that disorientation of prey is central to its envenomation strategy. Furthermore, venom expression profiles also suggest a sophisticated layering of venom-expression patterns within the venom duct, with disorientating and paralytic venoms expressed in different regions. Thus, our transcriptome analysis provides a new physiological framework for understanding the molecular envenomation strategy of this deadly snail.

## Background

Cone snails are venomous predators that rapidly immobilize their prey using a complex cocktail of short peptides (10–40 AA long) collectively known as conotoxins. Most of these peptides, are targeted with exquisite specificity to receptors, ion channels, and transporters in the nervous system [1-4]. Conotoxins are important pharmacological reagents and potential drug leads [5-8]. Each snail is believed to synthesize 50–200 such compounds [1,2,9], and with approximately 700 species [1,10], the venoms of the genus *Conus* constitute an exceptionally rich pharmacological resource.

We have analyzed the venom-duct of *Conus geographus* using a transcriptomics approach. This species, widely known as the geography cone, is well known as the deadliest of all cone snail species, responsible for most of the human fatalities recorded in the medical literature. In the absence of medical intervention, 70% of human stinging cases are fatal [11]. The venom of this cone snail species was the first that was comprehensively analyzed; it was the characterization of *Conus geographus* venom peptides which established that most of the biologically-active components of *Conus* venoms, were small, disulfide rich peptides [12].

In addition to being the deadliest of all cone snails, *Conus geographus* has an unusual strategy for catching fish: it is believed to prey primarily on schools of small fish hiding in reef crevices at night. It approaches potential prey with its false mouth highly distended, which is used as a net. It is believed to engulf multiple fish, and once the fish are enclosed within the gargantuan false mouth, it harpoons each fish, simultaneously injecting venom. The paralyzed prey are predigested within the false mouth, with the scales and bones of the fish regurgitated after 1–2 hours; the predigested soft parts of the fish are then moved further into the gut for complete digestion and absorption [13].

Several peptides from *Conus geographus* venom have become widely used in neuroscience research. There are several thousand citations in the scientific literature describing studies using ω-conotoxin GVIA, a specific inhibitor of Cav 2.2, a voltage-gated Ca channel subtype present at presynaptic terminus of many synapses (e.g. [14]). This peptide is widely used to study synaptic transmission. In addition, some venom peptides have therapeutic potential; one of them, conantokin G, a subtype specific NMDA receptor antagonist, selective for the NR2B subunit has reached human clinical trials as a potential drug for intractable epilepsy [15]. Thus, *Conus geographus* peptides are among the best characterized from any animal venom.

Although *Conus* venoms contain 100–200 different peptides, these are encoded by relatively few gene superfamilies (identified by capital letters). Previous transcript-based and proteomic studies have suggested that the *Conus* venom duct is a highly differentiated tissue, with anatomical and functional specialization along its length [16,17]. Garrett and coworkers [18], for example, investigated gene expression in the venom duct of *Conus textile*. To do so, they divided the venom duct into four sections, and used RT-PCR to profile the expression of toxins belonging to the A, O, M, T and P superfamilies using transcript-specific primers. Garrett *et al.* found that while all the superfamilies were abundantly represented in the proximal (P) segment, the expression of M, T, and P superfamilies declined progressively towards the distal end of the duct. In contrast, members of the O superfamily were highly expressed in distal portions of the duct.

Liquid chromatography/mass spectrometry and N-terminal sequencing analyses [18] have also suggested that conotoxin synthesis varies along the length of the duct. Tayo and coworkers [19], carried out a proteomic analysis of the regional distribution of conotoxins along the *Conus textile* venom duct using a tandem mass spectrometer to identify 31 conotoxin sequences and 25 posttranslational modifications. The abundance of most of these conotoxins varied among the different segments, with some toxins restricted to a single segment. An important observation from this study is that varying degrees of posttranslational modification of toxin molecules result in an overall variation of composition of the venom along the length of the duct. The cleavage site of the mature toxin from the propeptide was also found to vary along the length of the duct.

**Figure 1:**
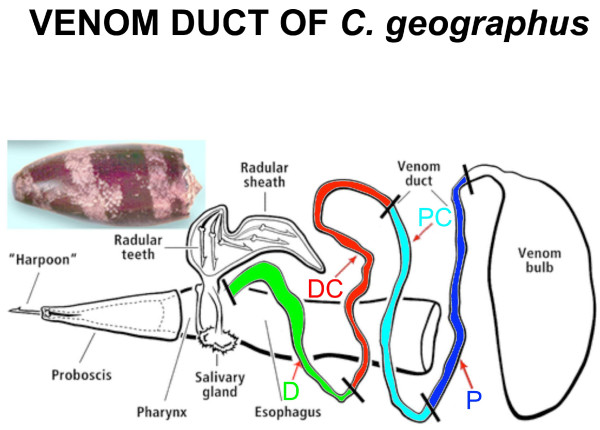
**The venom duct of *Conus geographus.* ** Insert: *Conus geographus* shell. Main figure: schematic of its venom duct. The segments of the venom duct are labeled as Proximal (P, in blue)--‒connected to the bulb, Proximal Central (PC, in purple), Distal Central (DC, in red), and Distal (D, in green) closest to the pharynx.

In order to more systemically investigate transcription within the venom duct, we have carried out large-scale RNA sequencing (RNA-seq) [20] in the same four regions (Figure 1) used by Garrett *et al.* in their PCR-based [18] and Tayo et al. [19] in their proteomics-based investigations of the venom duct of *Conus textile*. Our analyses complement and greatly extend their earlier work, providing a global and comprehensive overview of transcription along the length of the duct. Because the species of cone snail analyzed was *Conus geographus,* with well characterized toxins [3], some inferences about the frequency distribution of the transcripts can be made. We show that there exists clear transcriptional compartmentalization of the venom duct, with marked region-specific synthesis not only of conotoxins, but also for many other types of genes as well, such as insulin-like growth factors, which are highly expressed. The results suggest a potential role for these non-conotoxin genes in duct differentiation, physiology of venom delivery, or perhaps even as unrecognized components of the cone snail venom.

A unique aspect of the tissue being analyzed is that the venom duct of *Conus geographus* has arguably yielded more species-specific gene products than any other tissues in prior biochemical/functional analyses. The present transcriptome results, when correlated with this already substantial database, have provided surprising new insights into the physiology of envenomation by this remarkable snail that hunts fish and kills people.

## Results

### Transcriptome sequence datasets

Following dissection, the four segments of the venom duct of *Conus geographus* were prepared and sequenced independently. Using the Roche Genome Sequencer FLX Titanium platform, we generated 167,211, 238,682, 186,398 and 199,680 high-quality reads for the Proximal, Proximal-central, Distal-central and Distal segments, respectively. The average read length was 425.8 bp with an N50 read length of 580 bp.

### Transcriptome assembly

To generate a reference transcriptome for *Conus geographus,* we pooled the reads and then assembled them with Mira3 [21]. This generated 60,305 contigs totaling 34Mbp in length. We used cd-hit-est [22] to prune redundant contigs from the assembly arising from sequencing errors. See Methods for details. This produced a reference transcriptome assembly consisting of 49,515 contigs totaling 20.8 MB with an N50 of 576 bp. The median depth of coverage is 3.6x and the average depth is 26.7x. By aligning the raw reads in each segment back to the reference assembly with bwa [23], 98.7%, 99.3%, 99.1% and 99.2% of reads for the four segments aligned.

### Annotation

We annotated our reference transcriptome using BLASTX [24] and InterProScan [25]. A total of 8,252 contigs have a significant homology (BLASTX, E < 1e-4) to proteins in the Uniprot protein database [26] and/or the Conoserver conotoxin collection [27]. Among these 8,252 *geographus* contigs, 48.6% (4,010) are significantly homologous to known conotoxins.

InterProScan [25] search identified 5,420 contigs with protein domains, 2,216 of which (40.9%) are annotated as conotoxins. Among non-conotoxin contigs with InterProScan hits, the most abundant Gene Ontology [28] category is translation (GO:0006412) accounting for 1.1% of the total transcriptome assembly, followed by cellular iron ion hemostasis (GO:0006879), cell redox homeostasis (GO:0045454), electron transport chain (GO:0022900) and proteolysis (GO:0006508). The high level of transcripts related to translation and metabolism is consistent with venom duct physiology, as it is an organ engaged in intensive protein synthesis and processing [29,30]. This is also true for the GO terms related to redox homeostasis and proteolysis, as extensive post-translational modification of conotoxins as has been reported previously[19]. Current models of venom-duct physiology less easily explain the high level of iron ion hemostasis related GO-terms. Most of the transcripts falling into this GO category have significant homology to ferritin, which stores and regulates the release of iron. Further investigation will be required to explain the purpose underlying the high level of ferritin transcription in the duct.

### Functional overview of transcriptome assembly

**Figure 2:**
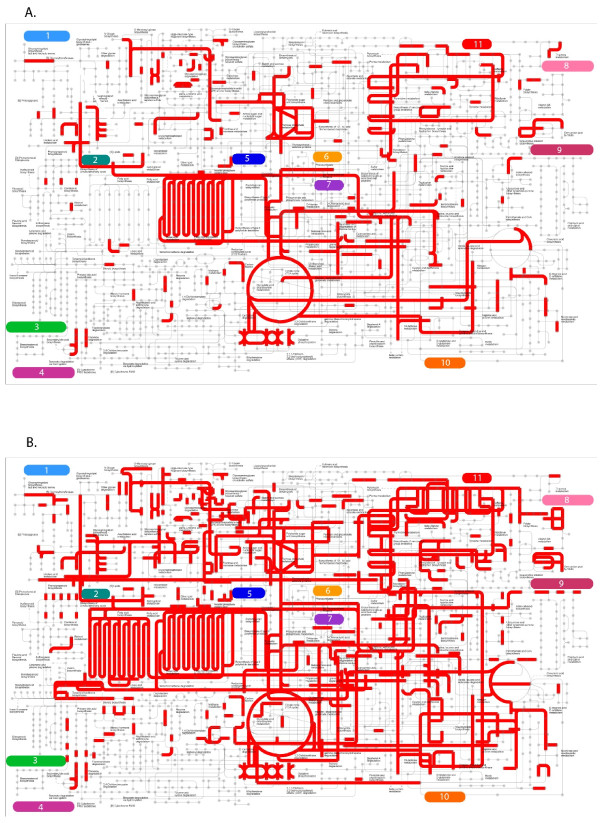
**iPath metabolism map.** (**a**) *Conus geographus*; (**b**) *Aplysia californica*. Each grey dot represents a metabolite and each red line represents an enzyme acting on it. Major pathways in the map includes: 1) Glycan biosynthesis and metabolism; 2) Lipid metabolism; 3) Metabolism of terpenoids and polyketides; 4) Xenobiotics biodegradation and metabolism; 5) Carbohydrate metabolism; 6) Amino acid metabolism; 7) Energy metabolism; 8) Metabolism of cofactors and vitamins; 9) Biosynthesis of other secondary metabolites; 10) Metabolism of other amino acid; 11) Nucleotide metabolism.

We used iPath explorer [31] to produce a high-level overview of the contents of our venom duct transcriptome (Figure 2A); see Methods for details. These provide a high-level summary of the metabolic, regulatory and secondary-metabolites biosynthesis pathways present within the transcriptome assembly (Additional file 1: Figure S1). We also generated the metabolic pathway map for *Aplysia californica*[32] (Figure 2B), using available all ESTs for this organism. As expected, we observed most components of the energy metabolism pathways in both maps, including the citrate cycle pathways, fatty acid metabolism pathways, carbohydrate metabolism pathways. However, by comparison to *A. californica*, the *C. geographus* venom duct transcriptome is less comprehensive, missing for example, 1) fatty acid synthesis pathways; 2) amino sugar metabolism pathways; 3) galactose metabolism pathways; 4) vitamin metabolism pathways and etc. These findings suggest that the above pathways are either absent from venom duct or at least being too inactive to be detected by RNA-seq approach, consistent with the duct having a highly streamlined metabolism geared at toxin secretion.

### Conotoxins are the most abundant class of transcript

Conotoxins comprised 48.6% of total annotated transcripts in the reference transcriptome or 88% of total aligned reads within annotated transcripts, suggesting that venom duct is a very high-throughput factory for the manufacture of conotoxins. To better understand the repertory of conotoxins present in the venom duct, we used a BLAST-based pipeline [33] to assign transcriptome contigs with homology to conotoxins to conotoxin superfamilies. In previous analyses [33] of the transcriptome in *Conus bullatus* this process is 98.7% accurate. In total, we were able to assign 1685 out of 4014 (42%) putative conotoxins a superfamily.

**Figure 3:**
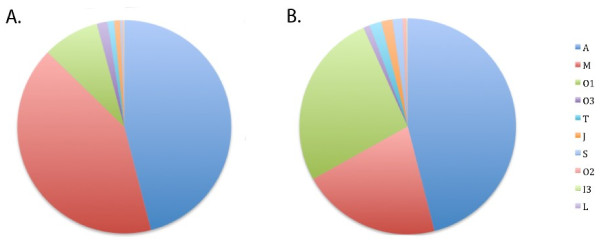
**Global relative - expression profiles of different conotoxin superfamilies.** Wedge - widths proportional to expression as measured by number of aligned reads. (**a**) and by number of contigs (**b**).

Figure 3 shows the proportion of each superfamily in the duct transcriptome, by reads (3a) and by contig numbers (3b). Consistent with Conoserver’s [27] contents, we found A-superfamily conotoxins to be the most abundantly expressed conotoxin superfamily in the *C. geographus* transcriptome assembly. However, while Conoserver currently only contains representatives from the A, M, O1, S and T superfamilies for *Conus geographus,* our transcriptome also contains J, O2, O3, I3 and Z conotoxins. For a list of toxins that we were able to retrieve full coding sequences, see Additional file 2: Table S1.

### Duct segments synthesize different spectra of conotoxins

Among the 49,515 contigs in our reference assembly, 3089 (6.2%) are differentially expressed among the four segments (*p* < 0.05/50,000 = 1E-6; see Methods for details). However, among conotoxin contigs, a much higher proportion are differentially expressed along the length of the duct: 1626 out of 3803 contigs or 42.8%. If we further restrict this analysis to conotoxin contigs composed of no fewer than 20 reads, thus giving us better statistical power to detect differential expression, the proportion increases to 87.5% (1501 out of 1715 conotoxin contigs). These results make it very clear that different segments of the duct may synthesize very different spectra of conotoxins.

To get a better understanding of the differences in expression profiles between the four duct segments, we first examined the Pearson correlation coefficient (r) between every pair-wise combination of duct segments using expression levels of homologous-protein-groups (HPGs; see Methods for details). These data are presented in Table 1. The best correlation is observed between the P and PC segments (R = 0.86); the worst are between P and D (R = 0.70) and PC and D (R = 0.72). All these correlations are statistically significant (*p* < 0.001), demonstrating that the general expression profiles between any two segments are correlated. In contrast, if we limit these comparisons to conotoxins alone, the profile of distal segment (D) is no longer significantly correlated with P and PC segment and only weakly correlated to DC (Table 1), indicating that as regards conotoxin expression, the distal segment expresses a distinct spectrum of conotoxins compared to the two proximal segments.

**Table 1:** Correlation of expression levels between segments

**a)**				
	**P**	**PC**	**DC**	**D**
P	**1**	**0.86**	**0.84**	**0.7**
PC		**1**	**0.84**	**0.72**
DC			**1**	**0.8**
D				**1**
**b)**				
	P	PC	DC	D
P	**1**	**0.86**	**0.77**	0.18
PC		**1**	**0.74**	0.11
DC			**1**	**0.48**
D				**1**

To further investigate the functional implications of these differences in segment expression profiles, we also examined the differential expression of InterPro protein families. Among 1356 protein families, 73 are differentially expressed, after Bonferroni correction (*p*-value < 0.05/1356) (See Additional file 3: Table S2 for the complete list). Translation-related proteins tend to be upregulated in PC. For example, the expression level of ribosomal proteins is 200% higher in PC compared to the other segments; in contrast, the expression levels of ribosomal proteins differ by less than 15% among the other three segments. Likewise, the level of translation initiation factor is 40% higher in the PC segment than the average of other three segments; and the level of translational elongation factors are 110% times higher in the PC segment compared to the others. These facts suggest that the PC is characterized by higher translational activity compared to the other three segments, and hence is capable of secreting greater volumes of venom. Consistent with these findings, we observed the highest expression of conotoxin transcripts in PC, 62% greater than the average of other three segments, as calculated by normalized read count.

We also discovered that a family of insulin-like factors (IPR016179) comprises an abundant class of duct transcripts. These transcripts are almost exclusively expressed in distal (D) segment (accounting for 99.6% of total insulin-like factors in the transcriptome overall). Moreover, insulin-like factors are the most abundant non-toxin IPR cluster in the distal segment. A closer examination of these transcripts reveals that they contain IGF insulin-like domains and at least some of them contain signal peptides (according to SignalP [34], D = 0.804, D-cutoff = 0.450). The presence of insulin-like factors in the venom duct has not been reported previously.

**Figure 4:**
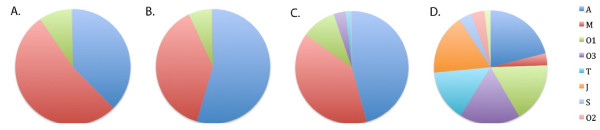
**Segment - specific relative - expression of conotoxinsuperfamilies.** Wedge - widths proportional to relative expression as measured by number of aligned reads. From **A** to **D**, Proximal (P), Proximal Central (PC), Distal Central (DC) and Distal (D) segments, respectively.

Figure 4 shows the makeup of conotoxin superfamilies in each segment. While P, PC and DC mostly express A, M and O1 superfamily conotoxins, segment D contains a much more diversified spectrum of conotoxins, including a significant proportion of T, J, O3, S, O2 and I3 superfamily conotoxins. These conotoxin families contribute to 0.3% of total conotoxin reads in segment P and PC, but make up 5.2% of segment DC and 58.4% of segment D.

**Figure 5:**
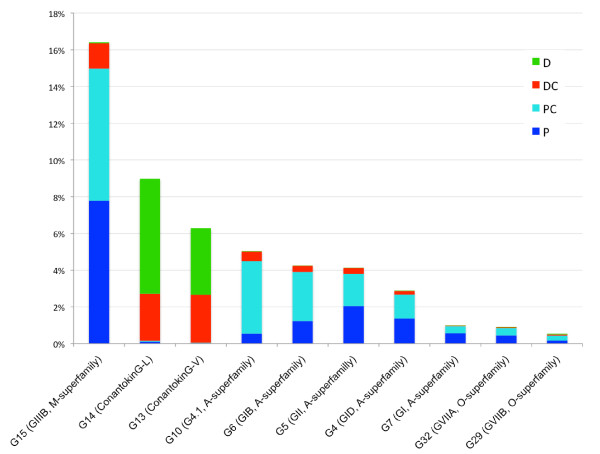
**Segment - specific expression profiles ofthe 10 most abundantconotoxin contigs.** Y - axis: total percent expression for the entire venom - duct broken down by segment. X - axis: toxin ID as in Additional file 2: Table S1, with its annotation and superfamily name shown in parentheses. Expression levels are scaled to the proportion of aligned reads among the whole transcriptome, divided into four segments: Proximal (P), Proximal Central (PC), Distal Central (DC) and Distal (D).

Analyses of the expression profiles of subclasses and individual conotoxins (Figure 5) reveal additional patterns of differential expression. For example, a Mu-conotoxins (G15), which are the most abundant conotoxin in the duct overall and accounting for 16.4% of all venom duct transcriptome reads, are predominantly expressed in the P and PC segments, with the DC segment accounting for only 8.3% of this 16.4%, and the D segment still less, containing 0.4% of all Mu-conotoxin reads. In contrast, 98.3% of ConantokinG-L (G14) reads are found in the two distal segments. This same pattern is also observed for ConantokinG-V. We also observed that for some conotoxin classes, the expression of different transcripts belonging to the same pharmacological family (e.g. alpha conotoxins) are highly correlated (data not shown) with one another from segment to segment. This finding may indicate that their expression is co-regulated.

## Discussion

We have used RNA-seq [20] to systematically investigate gene expression along the length of the venom duct of *Conus geographus*. Previous transcriptome studies of *Conus* species, including our own [33,35-38], have focused on conotoxins or post-translational modification enzymes. The RNA-seq analyses reported here are the first *for C. geographus*, and the first to investigate transcriptional gradients along the length of a *Conus* venom duct. These analyses have allowed us to assay the expression profiles of a wide spectrum of non-toxin as well as toxin transcripts. Gene Ontology analyses [28] of transcript abundance underscore the fact that the venom duct is a very metabolically active tissue expressing many genes associated with translation, as would be expected for a tissue manufacturing large numbers of conotoxins. GO analyses also identified high-levels of ferritin and other transcripts involved in control of iron and redox potential, indicating these facets of metabolism may be important for venom-duct metabolism.

We have also generated a metabolic pathway map for the venom duct by combining the RNA-seq datasets for each duct segment. Although likely incomplete, several interesting facts can be gleaned from it. For example, the venom duct possesses a complete complement of fatty acid metabolism pathways, suggesting it may use fatty acids as one of its main energy sources; going forward it will also be interesting to determine whether the absence of several vitamin and cofactor metabolism pathways is specific to venom duct, the whole animal, or merely an artifact of depth of coverage limitations in our RNA-seq datasets.

Our finding that there exist gradients of conotoxin gene expression along the length of the venom duct is consistent with previous reports. Studies by Garrett et al. [18] and Tayo et al. [19] on the venom duct of the mulluscivore *Conus texile* have shown that while A and O superfamilies and uniformly expressed, M and T superfamilies are mostly present in proximal and proximal central segments. Despite the fact that *Conus geographus* is a piscivorous cone snail, and is expected to synthesize a different combination of conotoxins, we do observe a few shared transcriptional profiles between these two species; for example, A and O superfamilies are expressed in all four venom duct segments in *Conus geographus,* and T superfamilies are mostly expressed in the two distal segments. Our RNA-seq approach, however, has allowed us to identify a far greater number of toxins families (Additional file 2: Table S1) and to characterize their patterns of differential expression (Figures 4 and 5) with much greater resolution.

Peptide toxins from *Conus geographus* venom have been more comprehensively characterized than for any other venom, but the transcriptomic analysis reported above provides significant new insights into the mechanism of prey capture by the snail. Shown in Table 2 are the major pharmacological sites targeted by highly-expressed conopeptides, as defined by the transcriptome analysis of *Conus geographus* venom. In several instances, multiple homologs were identified, probably allelic variants, almost certainly targeted to the same pharmacological site. The table shows the eight most frequent transcript classes targeted to specific sites, with all allelic homologs grouped together.

**Table 2:** Summary of the most highly expressed transcripts of toxins expressed in the venom duct

Pharmacological Sites Most Frequently Targeted	Rank of Site	Peptides Targeting Site	Sequence of Predicted Mature Venom Peptide	Total Reads	Distribution (% in quadrant)			
					P	PC	DC	D
NMDA Receptor (NR2B Subunit)	1	ConG	GEγγVQγNQγLIRγASNGKR	65,820	0.2	0.3	47.8	51.6
		ConG*	GEγγLQγNQγLIRγKSNGKR	90,531	0.7	0.4	34.4	64.6
Nav 1.4 (outer pore vestibule)	2	μ-GIIIA	RDCCTPPKKCKDRQCKPQRCCAGR	1,090	46.0	41.6	12.5	0.0
		μ-GIIIB	RDCCTPPRKCKDRRCKPMKCCAGR	110,980	41.2	43.2	15.0	0.5
Nicotinic AChR (Adult Muscle, αδ interface)	3	α-GI	ECCNPACGRHYSCGR	6, 431	50.7	40.4	8.6	0.4
		α-GIB	ECCNPACGRHYSCKGGR	29,256	24.8	60.8	13.9	0.4
		α-GII	ECCHPACGKHFSCGR	27,753	43.4	42.0	14.3	0.3
? Nicotinic AChR (Fetal Muscle, αγ interface)	4	αA(G4.1)	CCGKPNAACHPCVCNGSCSGRR	36,087	8.8	72.9	17.5	0.8
		αA(G4.2)	CCGKPNAACHPCVCNGSCSGGR	4,262	4.5	79.1	15.7	0.7
		αA(G4.3)	CCGKPTSACHPCVCNGSCSGGR	461	20.0	62.7	16.5	0.9
Neuronal nAChR (Subtype uncertain)	5	α-GID	ECCSNPACRVNNPHVCRRR	19,350	41.6	44.9	12.4	1.2
Calcium Channel (Subtype uncertain)	6	ωGVIIA ωGVIIB*	CKSPGTPCSRGMRDCCTSCLLYSNKCRRY	5,992	43.9	44.6	10.1	1.3
			CKSPGTPCSRTMRDCCTSCLSYSKKCRG	3,859	24.0	48.9	16.9	10.2
? Ca Channel(Unknown)	7	(G20)	CVPSGGSCSRTAYSCCHGSCSGGRCG	3,767	30.2	42.3	20.7	6.7
Calcium Channel (Cav2.2 subtype, outer vestibule)	8	ωGVIA	CKSPGSSCSPTSYNCCRSCNPYTKRCYG	3,611	61.0	18.6	11.3	9.2

The targets of the mature toxins, the total number of reads and their differential expression (percentage of the total) in the four segments are shown. * indicates allelic variants.

Not surprisingly, within the eight most highly ranked groups in Table 2 are components of *Conus geographus* venom that were the very first *Conus* peptides to be elucidated[3] . Two of these are known to cause neuromuscular paralysis in all vertebrates, and are probably the venom components responsible for the high rate of human fatality resulting from *Conus geographus* stings. The first group are antagonists of the muscle Na channel (μ-conotoxins GIIIA and GIIIB) and the second group are competitive nicotinic antagonists (α-conopeptides GI, GIB, and GII); both groups are very highly represented (112,070 and 63,440 reads, respectively). These two groups of peptides undoubtedly play a major role in disrupting neuromuscular transmission in the fish prey of *Conus geographus.* Somewhat surprisingly, the third type of conotoxin that disrupts neuromuscular transmission, the well-characterized presynaptic Ca channel inhibitor- ω-conotoxin GVIA (which is specifically targeted to Cav 2.2 voltage gated Ca channels), is represented in the transcriptome by a significantly lower level of mRNA (3,611 reads), although still one of the top eight in terms of rank of molecular targets. The other group of Ca channel targeted toxins, (ω-conotoxins GVIIA and GVIIB), which has not been quite as highly characterized, is known to also paralyze fish. These were present at a higher frequency than the ω-conotoxin GVIA, suggesting that they play a major role in inhibiting neuromuscular transmission.

The list of targeted pharmacological sites in Table 2 suggests that the nicotinic pharmacology of prey capture may be more complex than previously thought. One component, α-conotoxin GID is targeted to neuronal nicotinic receptors [39], but the precise endogenous physiological role for this peptide has not been elucidated. The fourth ranked molecular target in Table 2 is postulated to be the fetal form of the muscle nicotinic acetylcholine receptor [40]; none of the three peptides in this group have been isolated from *Conus geographus* venom. However, based on their predicted sequence they show high similarity to a peptide reported from *Conus obscurus*[40] (Additional file 4: Table S3), a small Conus species that is related to *Conus geographus*. By homology, we suggest that these three peptides (αA-conopeptides encoded by clones G 4.1, 4.2, and 4.3) are nicotinic antagonists specific for the fetal form of the receptor. Although the fetal muscle nicotinic receptor is expressed only very early in development in mammals, as was discussed previously, there is evidence that in fish it is expressed in adults [41]. Within the list of the top 8 molecular targets, another peptide G20 has never been isolated from Conus *geographus* venom either. It is highly likely, on the basis of its homology to ω-conotoxins GVIA and GVIIA, that this peptide is also targeted to voltage-gated Ca channels.

Thus, the transcriptome data have rounded the picture of the most frequent transcripts of this particular piscivorous cone snail species. The results of Table 2 suggest that the prey capture strategy of *Conus geographus* involves a more complex Ca channel pharmacology and targeting to a wider range of nicotinic receptors than was previously known.

The most unexpected facet of the results in Table 2 is that the most highly expressed group of transcripts encode two allelic variants of conantokin G, which is an N-Methyl-D-asparate (NMDA) receptor antagonist. It has been postulated that when *Conus geographus* approaches its fish prey, it releases some venom components to make the school of fish easier to capture, and that NMDA receptors in the lateral line circuitry of fish inhibited by conantokin G are a component of this strategy. These venom components, called the “nirvana cabal,” comprise a group of peptides that cause hypoactivity and disorientation in the fish even before they are stung (2 already listed). The very high frequency of these transcripts suggests that this is an important component in the prey capture strategy of *Conus geographus*. All of the peptides above were purified from venom in the initial characterization of *Conus geographus* venom peptides [9].

Our results make it clear the venom duct is a high-throughput factory for the manufacture of conotoxins—over 48% of the annotated transcripts in our reference transcriptome are homologous to conotoxins, and these account for 88% of reads aligning to annotated transcripts. We have also discovered that different portions of the duct have different toxin-expression profiles. Among the 63 conotoxin transcripts listed in Additional file 2: Table S1, 9 are uniquely expressed in D (P, PC and DC express 1, 1 and 0 unique conotoxins, respectively). Moreover, while the other segments predominantly express conotoxins belonging to the A, M and O1 superfamilies, the D segment expresses a significant proportion of T, J, O3, S, O2 and I3 superfamily conotoxins. These conotoxin families, for example, contribute to only 0.3% of total conotoxin reads in the P and PC segments, but comprise 58.4% of the distal D segment’s transcripts. These facts, combined with the observation that the normalized average expression level of conotoxin transcripts is lower than any other segment (e.g. half that of PC), raised the possibility that the D segment may be a specialized production site for a wide spectrum of the less abundant peptide toxins.

We also discovered that insulin-like growth factors are highly expressed in the distal (D) segment, comprising the most abundant non-toxin transcript in this segment. As regards the function of these IGF-like transcripts, two hypotheses suggest themselves. First, the proteins encoded by these transcripts might serve as growth factors to stimulate cell proliferation. Given the unique expression profile of the D segment, and the fact that these IGF-like transcripts were not detected in the proximal portions of the duct, these proteins may well play a region specific role in differentiation of the distal portions of the duct. Also intriguing is the possibility that IGF proteins might play a function in venoms. High concentrations of IGF-like proteins have been shown to activate the insulin receptor and to mimic the effects of insulin [42-44]. Given that *Conus geographus* is a fish-hunting snail, these IGF-like proteins might be used as part of the strategy to disorient prey. Further functional analyses of course will be required before either hypothesis can be afforded a status beyond mere speculation. In any case, the differential expression of these transcripts along the length of the duct provides further support for our central conclusion, namely that the venom duct contains specialized compartments along its length, each synthesizing a distinctive spectrum of venom components.

## Conclusion

We manufactured RNA-seq libraries for four different regions along the length the *Conus geographus* venom duct. We have identified in these datasets, 37 novel conotoxins belonging to seven different superfamilies (A, I, S, T, O, J and con-ikot-ikot). These are potentially new reagents for neurobiological research and pharmaceuticals. Our analyses also demonstrate the existence of region-specific differences in gene-expression along the length of the duct, with clear proximal-distal differences both as regards general gene-expression profiles as well as venom genes. Moreover gene expression profiles in the distal portion of the duct differ dramatically from other regions. While the other segments predominantly express conotoxins belonging to the A, M and O1 superfamilies conotoxins, the distal segment expresses high levels of conotoxins belonging to the T, J, O3, S, O2 and I3 superfamilies. These differences suggest the existence of still further sub-compartmentalization along the length of the duct. We also discovered high levels of insulin-like growth factors in the duct, again expressed almost exclusively in distal central section of the duct.

The results shed new light on the prey capture strategy of *C. geographus*. The very high expression of conantokins, NMDA receptor antagonists which cause sluggishness and disorientation, underscores their role in prey capture. As expected the toxins involved in neuromuscular paralysis, the Na channel (μ-conotoxin) and nicotinic acetylcholine receptor antagonists(α-conotoxin) are highly expressed in the venom duct. However, the high expression of ω-GVIIA and ω-GVIIB suggest their hitherto unrecognized importance as Ca channel antagonists in prey capture. In addition, the high expression levels of α-Conotoxin GID, novel αA conotoxins G4.1, 4.2 and 4.3, and the ω-contoxin G20 imply their involvement in prey capture, and make the need to identify their specific targets more urgent.

## Methods

### Sample collection and preparation

Specimens of *Conus geographus* were collected in Cebu province, Philippines. The snails were 10 cm to 14 cm in length and the ducts 17 cm to 24 cm in total length. Immediately after collection the snails were placed in ice and dissected. The venom duct was divided into four equal-length segments and stored in RNA later® (Applied Biosystems, Inc). The corresponding segments from four animals were pooled for the isolation of RNA. Total RNA was isolated using Trizol® Plus RNA purification kit (Invitrogen, life Technologies) and the quality of RNA was determined using the Agilent Bioanalyzer, with the RNA integrity number (RIN) being between 7 and 8. The RNA samples were then sent to 454 Life Sciences, and the cDNA preparation and sequencing steps were performed in-house.

### Transcriptome assembly

To maximize the breadth of the transcriptome assembly, reads sequenced from different segments were pooled prior to assembly. We followed the assembly instructions in the Mira manual [45], and found that the following options optimized the assembly as regards continuity and minimal numbers of chimeric transcripts: -job = denovo,est,normal,454 -GE:not = 15 COMMON_SETTINGS -CL:ascdc = 1 -SK:not = 15 -AS:nop = 4 454_SETTINGS -ED:ace = 1 -AS:mrl = 30:mrpc = 1 -OUT:sssip = yes -CL:qc = 0:cpat = yes. Upon manual inspection of the assembly, we identified some mRNA species that were assembled as multiple contigs because of sequencing errors. This could be an issue for differential expression analyses since the power of detecting differential expression will be decreased if reads belonging to the same transcript are mapped to multiple contigs. To control for this effect, we used cd-hit-est [22] to cluster redundant transcripts and generated a set of representative ESTs (parameter: -M 0 -T 8 -c 0.95 -r 1). By aligning raw reads to the resulting representative set with bwa-sw [23] (using default parameters), 99% of total reads aligned, confirming that the quality of assembly is good. We thus designate this assembly as our reference transcriptome assembly.

### Generation of pathways maps

We used the KEGG Automatic Annotation Server [46] to associate KEGG orthology (KO) terms to contigs in the reference assembly. The SBH method was chosen to ensure a high sensitivity . The KO annotations were then uploaded to iPath server [31] to generate the pathway maps. To generate the *Aplysia californica* pathway maps, we downloaded the comprehensive EST set for this species from UCSC genome browser [32] and applied the same procedures. By this approach some components of metabolism pathways may be missing from the map if they are expressed at very low levels in the venom duct.

### Correlation of expression profiles

For each contig in our reference transcriptome assembly, we counted the number of reads from each segment that aligned. For the correlation analyses, the contigs are grouped based on their best homologous sequences (using BLASTX) in the Uniprot protein database or Conoserver toxin collection. This allows us to combine non-overlapping contigs sharing the same best protein hit. The numbers of reads in each homologous-protein-group (HPG) were then calculated by summing the numbers of reads aligned to each contig in this group. These counts were converted to log_10_ scale (log normalized) to avoid bias toward very highly expressed transcripts. We then calculated Pearson’s correlation coefficient (R) using the read counts for each HPG between every two segments. The significance levels for these correlations were calculated by permutation.

### Differential expression analysis

A widely used statistic for differential expression is FPKM value (Fragments Per Kb exon model per Million mapped reads) [47], which normalizes expression for both library size and transcript length. However, one shortcoming of the FPKM approach is that if a few very highly expressed transcripts are differentially expressed across samples, the expression level of other transcripts as represented by the FPKM value can be distorted leading to false-positives and false-negatives. This is the case for our analyses, as a few conotoxins (e.g. Mu-conotoxin) dominate the venom duct-transcript counts for some of the segments. In such situations, one alternative to FPKM is to use a house-keeping gene for normalization, but here the concern is that using just one house-keeping gene may lead to inaccurate normalizations. Thus, we used a more appropriate approach for normalization. The trimmed mean of M-values method as developed by Mark D. Robinson and Alicia Oshlack [48] uses the median proportion of all expressed genes rather than a few housekeeping genes for normalization. This has shown to dramatically improve results on both simulated and real datasets [48]. We therefore used this method in our differential expression analyses. The details of the algorithm are available in the original article. Briefly, an M-value (empirical measurement of observed vs. expected expression level) is first calculated for every gene included in the comparison. After removing genes with top/bottom tiers of M-values and expression levels, the ratio of RNA product level of two samples is calculated based on remaining genes. This ratio was then used to normalize the library size (total number of mapped reads) for each segment. Manual examination of the expression level of a group of housekeeping genes provided further support that this normalization approach was appropriate.

Since we are comparing four biological samples without replicates, a chi-square test based on normalized library size is most appropriate for differential expression analyses. Therefore, for each contig in our reference transcriptome, we used a chi-square test to calculate *p*-values under the null hypotheses that proportions of this transcript among the cDNA library are the same across the four segments. In cases where the expected reads in any cell is below 5, we instead used the fisher-exact test to calculate the *p*-value. The threshold for statistical significance was set to 0.05/49515 = 1e-6, to control for multiple tests. For the differential expression of HPG or InterPro protein families, the same procedures are applied and Bonferroni-corrected alpha were set according to the number of comparisons performed.

### Data availability

The sequence data from this study are submitted to the NCBI Sequence Read Archive (http://www.ncbi.nlm.nih.gov/Traces/sra/sra.cgi) under accession numbers SRR503413, SRR503414, SRR503415 and SRR503416.

## Abbreviations

P, Proximal; PC, Proximal-central; DC, Distal-central; D, Distal; HPG, Homologous protein group; NMDA, N-Methyl-D-asparate; IGF, Insulin growth factor; FPKM, Fragments Per Kb exon model per Million mapped reads.

## Competing interests

The authors declare that they have no competing interests.

## Authors’ contributions

HH designed, performed the bioinformatic analysis and drafted the manuscript. PB prepared cDNA samples for sequencing, analyzed conotoxin sequences and drafted the manuscript. MY and BO conceived, supervised the study and draft the manuscript. All authors read and approved the final manuscript.

## Supplementary Material

Additional file 1: Figure S1. **iPath (a) regulatory pathway map and (b) secondary-metabolite biosynthesis pathway map.** Each grey dot represents a substrate and each red line represents an enzyme. Click here for file

Additional file 2: Table S1. **A list of complete conotoxins sequences identified in the venom duct.** The expression levels are shown for each conotoxin in each segment, represented as number of reads aligned to the toxin. Toxins are numbered G1-63, and have been listed according to their superfamilies. In the A superfamily, (X,Y) refers to the number of amino acid residues in the first and second disulfide loops. G4.x is the commonly used nomenclature of alpha-A family of conotoxins identified from *Conus geographus.* Other designations in parenthesis adjacent to G**X** indicate previously used nomenclature in the literature [3,4,12,49-55]. Click here for file

Additional file 3: Table S2. **InterPro protein families differentially expressed among the four segments Proximal (P), Proximal Central (PC), Distal Central (DC) and Distal (D), showing the proportion of aligned reads among whole transcriptome [**[3]**,**[4]**,**[12]**,**[49-55]**].** Click here for file

Additional file 4: Table S3. **Comparison of toxin sequences of αA-OIVA, a fetal muscle nicotinic acetylcholine receptor antagonist, with G10 4.1 and G11 4.2 [**[3]**,**[4]**,**[12]**,**[49-55]**].** Click here for file
