# Supplementary material for: Elucidation of the molecular envenomation strategy of the cone snail Conus geographus through transcriptome sequencing of its venom duct
Source: BMC Genomics. 2012 Jun 28;13:284. doi: 10.1186/1471-2164-13-284 (PMC3441800; doi:10.1186/1471-2164-13-284)
Supplement: Additional file 1: Figure S1. — iPath (a) regulatory pathway map and (b) secondary-metabolite biosynthesis pathway map. Each grey dot represents a substrate and each red line represents an enzyme. [file 1471-2164-13-284-S1.doc]

**Supplemental Table 1.** A list of complete conotoxins sequences identified in the venom duct. The expression levels are shown for each conotoxin in each segment, represented as number of reads aligned to the toxin. Toxins are numbered G1-63, and have been listed according to their superfamilies. In the A superfamily, (X,Y) refers to the number of amino acid residues in the first and second disulfide loops. G4.x is the commonly used nomenclature of alpha-A family of conotoxins identified from *Conus geographus.* Other designations in parenthesis adjacent to G**X** indicate previously used nomenclature in the literature.

| Classification | | Differential | | | Novel toxin | | | P | PC | | DC | | | D | |  |
| --- | --- | --- | --- | --- | --- | --- | --- | --- | --- | --- | --- | --- | --- | --- | --- | --- |
| **A-superfamily** | |  | | |  | | |  |  | |  | | |  | |  |
| **(4,7)** | |  | | |  | | |  |  | |  | | |  | |  |
| G1 | | yes | | | no | | | 6 | 5 | | 4 | | | 648 | |  |
| MGMRMMFTVFLLVALATTVVSFTSDRASDRRNAAVKAFDLISSTVKKGCCSHPACSGNNPEYCRQGR | | | | | | | | | | | | | | | |  |
| G2 | | yes | | | no | | | 8 | 3 | | 30 | | | 953 | |  |
| MGMRIMFTVFLLVALATIVVSFTSDRASDGRNAAAKAFDLKGCCSHPACSGNYQEYCRESY | | | | | | | | | | | | | |  | |  |
| G3 | | yes | | | yes | | | 150 | 43 | | 10 | | | 4 | |  |
| MGMRMMFTVFLFLVLATTDVSFTLDRASDGGNAAAKKSAVIALSRRRCCGIKSCYENNKDMCGRRR | | | | | | | | | | | | | | | |  |
| G4(GID, [1]) | | yes | | | no | | | 8040 | 8684 | | 2395 | | | 231 | |  |
| MGMRMMFTVFLLVVLAATIVSFTSDRASDGRNVAAKAFHRIGRTIRDECCSNPACRVNNPHVCRRR | | | | | | | | | | | | | | | |  |
| **A-superfamily**  **(3,5)** | |  | | |  | | |  |  | |  | | |  | |  |
| G5(GII,[2]) | | yes | | | no | | | 12039 | 11665 | | 3958 | | | 91 | |  |
| MGMRMMFTVFLLVVLATTVVSFPSERASDGRDDTAKDEGSDMDKLVEKKECCHPACGKHFSCGR | | | | | | | | | | | | | | | |  |
| G6(GIB,[2]) | | yes | | | no | | | 7258 | 17791 | | 4077 | | | 130 | |  |
| MRMMFTVFLLVVLATTVVSFPSERASDGRDDTAKDEGSDMEKLVEKKECCNPACGRHYSCKGGR | | | | | | | | | | | | | | | |  |
| G7(GI,[2]) | | yes | | | no | | | 3259 | 2596 | | 553 | | | 26 | |  |
| MGMRMMFTVFLLVVLATTVVSFPSERASDGRDDTAKDEGSDMDKLVEKKECCNPACGRHYSCGR | | | | | | | | | | | | | | | |  |
| **A-superfamily**  **(5,10)** | |  | | |  | | |  |  | |  | | |  | |  |
| G8 | | yes | | | yes | | | 335 | 2049 | | 725 | | | 24 | |  |
| MGMRMMFTVFLLVVLATTVVSSTSDRASDGRNAAASDKASDLITQVVKRCCGKPNDCLPSFAFVNGSCNGRR | | | | | | | | | | | | | | | |  |
| **A-superfamily**  **(4,8)** | |  | | |  | | |  |  | |  | | |  | |  |
| G9 | | yes | | | no | | | 106 | 51 | | 855 | | | 48 | |  |
| GMRMMFTVFLLVVLATTVVSFTSRRGPKSRRGEPVPTTVINYGECCKDPSCWVKVKDFQCPGASPPN | | | | | | | | | | | | | | | |  |
| **α-A superfamily** | |  | | |  | | |  |  | |  | | |  | |  |
| G10(G4.1) | | yes | | | yes | | | 3160 | 26316 | | 6316 | | | 295 | |  |
| MGMRMMFTVFLLVVLATTVVSSTSDRASDGRNAAASDKASDLITQVVKRCCGKPNAACHPCVCNGSCSGRR | | | | | | | | | | | | | | | |  |
| G11(G4.2) | | yes | | | yes | | | 92 | 289 | | 76 | | | 4 | |  |
| MGMRMMFTVFLLVSTWQRLSFPPLQIVHLMAGMPQASDKASDLITQVVKRCCGKPTSACHPCVCNGSCSGGR | | | | | | | | | | | | | | | |  |
| G12(G4.3) | | yes | | | no | | | 190 | 3370 | | 671 | | | 31 | |  |
| MGMRMMFTVFLLVAWQRLVSSTSDRASDGRNAAASDKASDLITQVVKRCCGKPNAACHPCVCNGSCSGGR | | | | | | | | | | | | | | | |  |
|  | |  | | |  | | |  |  | |  | | |  | |  |
| **Conantokin** | |  | | |  | | |  |  | |  | | |  | |  |
| G13(ConG-V,[3]) | | yes | | | no | | | 156 | 193 | | 31489 | | | 33982 | |  |
| MHLYTYLYLLVPLVTFHLILGTGTLDDGGALTERRSADATALKAEPVLLQKSAARSTDDNGKDRLTQMKRILKQRGNKARGEEEVQENQELIREASNGKR | | | | | | | | | | | | | | | |  |
| G14(ConG-L,[3]) | | yes | | | no | | | 595 | 325 | | 31098 | | | 58513 | |  |
| MHLYTYLYLLVPLVTFHLILGTGTLDDGGALTERRSADATALKAEPVLLQKSAARSTDDNGKDRLTQMKRILKQRGNKARGEEELQENQELIREKSNGKR | | | | | | | | | | | | | | | |  |
| **M-superfamily** | |  | | |  | | |  |  | |  | | |  | |  |
| G15(GIIIB,[4]) | | yes | | | no | | | 45772 | 47983 | | 16622 | | | 603 | |  |
| MMSKLGVLLTICLLLFPLTALPMDGDEPANRPVERMQDNISSEQYPLFEKRRDCCTPPRKCKDRRCKPMKCCAGR | | | | | | | | | | | | | | | |  |
| G16(GIIIA,[4]) | | yes | | | no | | | 501 | 453 | | 136 | | | 0 | |  |
| NISSEQYPLFEKRRDCCTPPKKCKDRQCKPQRCCAGR | | | | | | | | | | | | | | | |  |
| **O-superfamily** | |  | | |  | | |  |  | |  | | |  | |  |
| G17 | | yes | | | yes | | | 125 | 58 | | 16 | | | 14 | |  |
| MKLTCVVIVAVLLLTACQLIAALDSRGMLKHRALRSTKVSKSPPCLVAGSSCRGTTRVCCGFCSHYGYKCRDRPTS | | | | | | | | | | | | | | | |  |
| G18 | | yes | | | yes | | | 312 | 569 | | 695 | | | 316 | |  |
| MKLTCVVIVAALLLTACQLITALDCGGTQKHRALRSTIKLSLLRQHRGWCGDPGATCGKLRLYCCSGFCDC | | | | | | | | | | | | | | | |  |
| G19 | | yes | | | yes | | | 113 | 253 | | 37 | | | 168 | |  |
| MKLTCVVIVAALLLTACQLITALDCGGTQKHRALRSTIKLSLLRQHRGWCGDPGATCGKLRLYCCSGFCDCYTKTCKDKSSA | | | | | | | | | | | | | | | |  |
| G20 | | yes | | | yes | | | 1138 | 1594 | | 781 | | | 254 | |  |
| MKLTCVVIVAVLLLTACQLITADDSRGTQKHRALRSSTKLTLSTRCVPSGGSCSRTAYSCCHGSCSGGRCG | | | | | | | | | | | | | | | |  |
| G21 | | yes | | | yes | | | 4 | 7 | | 534 | | | 1203 | |  |
| MSGLRIMVLTLLLLVLMTTSHQDAGEKQAMQRDAKNFSRRRLGIRKPKTRECEMLCEQEEKHCCRIRNENIQCAPRCLGIGV | | | | | | | | | | | | | | | |  |
| G22 | | yes | | | yes | | | 96 | 127 | | 43 | | | 34 | |  |
| MNLTCVLIINVLFLTACQVITADDSRDKQIYRAVRSRDGMRNFRSSRPCANLGRACDTVPCCLGVRCFESRTPTCLLKQRGV | | | | | | | | | | | | | | | |  |
| G23(C.geographus-GS-A) | | yes | | | no | | | 257 | 1069 | | 240 | | | 9 | |  |
| MNLTCVLIIAVLFLTACQLIAADDSRDNQKHRAVRMRDALKNFKDSRACSGRGSRCPPQCCMGLTCGREYPPRCG | | | | | | | | | | | | | | | |  |
| G24 | | yes | | | yes | | | 3 | 10 | | 117 | | | 236 | |  |
| MNLTCVLIIAVLFLTTCQLITADDSRDKQKYRAVRLGDEIQIFKTRRRRCVGRDSKCGPPPCCMGMTCNYERVRKCT | | | | | | | | | | | | | | | |  |
| G25 | | yes | | | yes | | | 1 | 0 | | 13 | | | 27 | |  |
| MNLTCVLIIAVLFLTACQLIAADDSRDKQKYRAVRLGDEMQIFKTREKLCGELYDGCHDQRCCPGLTCDTLFQCVRHS | | | | | | | | | | | | | | | |  |
| G26(G6.8) | | yes | | | no | | | 0 | 0 | | 59 | | | 487 | |  |
| MEKLTILLLAAAVLMSTQATIQGGGENRPKENIKYLSKSQRSAERGVWSECSDWLAGCSSPSECCSEKCDTFCRLWR | | | | | | | | | | | | | | | |  |
| G27 | | yes | | | yes | | | 91 | 261 | | 30 | | | 77 | |  |
| MSGLGIMVLTLLLLVSMAISHRYAREKQATRRDVVNIRRRSKPKTPECKRICKLEEKKCCCVRSEGPKCSRLCGLPMFC | | | | | | | | | | | | | | | |  |
| G28(GVIA, [5]) | | yes | | | no | | | 2203 | 670 | | 407 | | | 331 | |  |
| MKLTCVVIVAVLLLTACQLITTEDSRGTQKHRALGSTTELSLSTRCKSPGSSCSPTSYNCCRSCNPYTKRCYG | | | | | | | | | | | | | | | |  |
| G29(GVIIB*) | | yes | | | yes | | | 927 | 1886 | | 653 | | | 393 | |  |
| MKLTCVVIVAVLLLTACQLITADDSRGTQKHRALRSSTKLTLSTRCKSPGTPCSRTMRDCCTSCLSYSKKCRG | | | | | | | | | | | | | | | |  |
| G30(Conotoxin GS, [6]) | | yes | | | no | | | 8 | 10 | | 451 | | | 11 | |  |
| MKLTCLLIIAVLFLTACQLITANDPRDNQEYRAVRMKDALNFKDSRACSGRGSRCPPQCCMGLRCGRGNPQKCIGAHEDV | | | | | | | | | | | | | | | |  |
| G31 | | no | | | yes | | | 0 | 0 | | 0 | | | 1 | |  |
| MKCTVLLLISGVISCNDKQKYRAVRLGDEMQIFKTREKLCGELYDGCHDQRCCPGLTCDTLFQCVRHS | | | | | | | | | | | | | | | |  |
| G32(GVIIA,  [7]) | | yes | | | no | | | 2632 | 2675 | | 607 | | | 78 | |  |
| MKLTCVVIVAVLLLTACQLITADDSRGTQKHRALRSSTKLTLSTRCKSPGTPCSRGMRDCCTSCLLYSNKCRRY | | | | | | | | | | | | | | | |  |
| G33 | | no | | | yes | | | 0 | 0 | | 0 | | | 1 | |  |
| MKLTCALIVAVLFLMACQLITAENSRGSREYSAVRSSDKIRDSDDRQLTKECTQDFDPCMPVCHECCTRSHFVVCRRPICLRRIF | | | | | | | | | | | | | | | |  |
| G34 | | yes | | | yes | | | 11 | 0 | | 159 | | | 326 | |  |
| MKLTCMMIVAVLFLTAWTFVTAEDSRDARTPMCGATCAMACPNDFVEDEHGCPICKCREEKRTDETNLV | | | | | | | | | | | | | | | |  |
| G35 | | no | | | yes | | | 0 | 0 | | 0 | | | 3 | |  |
| MKLTCVVIVAVLLLTACQLITALDSRGTQKHHALRSTTKLSMLRTSRDWCGDAGDACGTLKLRCCSGLCNQYSGTCTG | | | | | | | | | | | | | | | |  |
| **T-superfamily** | |  | | |  | | |  |  | |  | | |  | |  |
| G36 | | yes | | | yes | | | 0 | 0 | | 0 | | | 88 | |  |
| MLCLRVLIILLLLASPAASNPLETRIQSDLIRAALEDADIKTEKGFLGSVFSNLGGITDLATGICCAIIERCCVK | | | | | | | | | | | | | | | |  |
| G37 | | yes | | | yes | | | 13 | 89 | | 443 | | | 1475 | |  |
| MPCLPVFIILLLLISSAPSVDARPKTKYDVSRASFHVNAKRDRQSRWMERDCCEERWCCFR | | | | | | | | | | | | | |  | |  |
| G38 | | yes | | | yes | | | 0 | 0 | | 0 | | | 49 | |  |
| MQCLPVFTILLLLASTAAPNPLETRIQSDLTRADLEDSDTKTDERFITGLLGGLSAVGGITSLASRICCAITDSCC | | | | | | | | | | | | | | | |  |
| G39 | | yes | | | no | | | 0 | 0 | | 0 | | | 179 | |  |
| MLSLPVFIILLLLASPAAPIPLETRIQSDLIRAALEDADMKNEKGLLNGLVGNLGEIGEIISTVCCSVYPKCCVEK | | | | | | | | | | | | | | | |  |
| G40 | | yes | | | no | | | 0 | 0 | | 0 | | | 17 | |  |
| MLCLPVFIILLLLASPAAPKPFETKLPSDLTRADVDIDMAVFLEKLQDACCKNAPEFGCCTR | | | | | | | | | | | | | | | |  |
| G41(Tx5.2,[8]) | | yes | | | no | | | 13 | 89 | | 53 | | | 352 | |  |
| MCCLPVFVILLLLITSAPSVDALPKTRDDVPLASFHGGYNARRILQRRQGWCCKENIACCI | | | | | | | | | | | | | | | |  |
| **S-superfamily** | |  | | |  | | |  |  | |  | | |  | |  |
| G42(GVIIIA, [9]) | | yes | | | no | | | 663 | 205 | | 361 | | | 781 | |  |
| MMSKMGAMFVLLLLFTLASSLQEGDVQARKTRLKSDFYRALARDDRGCTRTCGGPKCTGTCTCTNSSKCGCRYNVHPSGWGCGCACSG | | | | | | | | | | | | | | | |  |
| G43 | | yes | | | yes | | | 4 | 2 | | 7 | | | 57 | |  |
| MMSKMGAMFDLLLLFTLASSQQEGDVQARKTRLKSGFHRALAMDDRGCTRTCGGADCTGSCECTFSSNCGCEFHGGPGAWGCACVCSG | | | | | | | | | | | | | | | |  |
| G44 | | yes | | | yes | | | 49 | 17 | | 7 | | | 25 | |  |
| MMSKMGAMFVLLLLFTLASSQQEGDVQARKTRPKSDFYRALPRSGSTCTCFTSTNCQGSCECLSPPGCYCSNNGIRQRGCSCTCPGTG | | | | | | | | | | | | | | | |  |
| G45 | | yes | | | yes | | | 0 | 0 | | 0 | | | 2 | |  |
| MSRMGVVCFLLLLFTLASTRQEGDAEARKIDNLNNRHAFLLWADGDCTCGKTSSCRTGTCGCRGSNCKCERGVYRSAEKCHCVCPQ | | | | | | | | | | | | | | | |  |
| G46 | | yes | | | yes | | | 55 | 2 | | 184 | | | 516 | |  |
| MMSKMGAMFVLLLLFTLASSLQEGDVQARKTRLKSDFYRALADVGECTHCGGADCTGSCTCTNWSSCVCKYFSSSGAGECGCACYD | | | | | | | | | | | | | | | |  |
| **Contryphan** | |  | | |  | | |  |  | |  | | |  | |  |
| G47(Contryphan-G) | | yes | | | no | | | 7 | 1 | | 16 | | | 161 | |  |
| MGKLTILVLVAAVLLSTQAMVQGDGDQPAARNAVPRDDNPDGPSAKFMNVQRRSGCPWEPWCG | | | | | | | | | | | | | | | |  |
| **I-superfamily** | |  | | |  | | |  |  | |  | | |  | |  |
| G48 | | yes | | | yes | | | 0 | 0 | | 4 | | | 287 | |  |
| MKLFLAIVLILMLLSLSTGAETSDNHASRSATALRDWLLGPLAKRCAVTHEKCSDDYDCCGSLCCVGICAKTIAPCK | | | | | | | | | | | | | | | |  |
| **J-superfamily** | |  | | |  | | |  |  | |  | | |  | |  |
| G49 | | yes | | | yes | | | 0 | 0 | | 0 | | | 153 | |  |
| MTSVQSVTCCCLLWLMLSVQPITPGSPGPAQLSRERSFRFIPGGIKEIACHRYCAKGIASAFCNCPDKRDVVSPRIRRRKRSKAM | | | | | | | | | | | | | | | |  |
| G50 | | yes | | | yes | | | 3 | 3 | | 1 | | | 304 | |  |
| MTSVQSVTCCCLLWLMLSVQPITPGSPGPAQLSRERSFRFIPGGIKEIACHRYCAKGIASAFCICPDKRDVVSPRIRRRKRSKAM | | | | | | | | | | | | | | | |  |
| G51 | | no | | | yes | | | 3 | 0 | | 0 | | | 0 | |  |
| MPSVQSVTRCCLLWLMLSVQLVTPGSRGTAQLPRDDLDETTREEICRYMCSRKIDYHMCTCPSKRDAISSRIVRRKRSMAV | | | | | | | | | | | | | | | |  |
| G52 (Scratcher related, [10]) | | yes | | | no | | | 1 | 0 | | 2 | | | 2034 | |  |
| MTSVQSVTCCCLLWLMLSVQPITPGSPGPAQLSRERSFKFLSGGFKEIVCHRYCAKGIAKEFCNCPDKRDVVSSRIRGRKRSKAM | | | | | | | | | | | | | | | |  |
| **Con-ikot-ikot** | | | | | | | | | | | | | | | |  |
| G53 | | yes | | | yes | | | 2 | 1 | | 2335 | | | 1328 | |  |
| MAMSMSMTLSVFVMVVMAATVTGFTQLKKPDLSRMKRNNKVCCNQRNLPMSEAEILDKKVITLTHVNKKAVTSCPGSDIDGCCPGYTMCMSTNAQNNVHTAHTSCLNRPCFGPCK | | | | | | | | | | | | | | | |  |
| G54 | | yes | | | yes | | | 3 | 1 | | 908 | | | 2241 | |  |
| MAMNMSVMLSAFVMVVVSATVTGFTHLQEPDLSRMERSPPPHNDCCKMKECCAQTTELCLKEFPNEEHIYTSTCYQRASHACGQFNEIVGCCYGYRQCMLQNVQNLGLNWANQQCKEWNCLNPCE | | | | | | | | | | | | | | | |  |
| G55 | | yes | | | yes | | | 0 | 0 | | 461 | | | 1060 | |  |
| MAMSMSMTLSVFIMGVVAATVTGFTDLQVPNLSRMERNEPLDCCNMKTCCVRSMYECLQRHPGNENNMVSSCYHEAGDICGSYNEIVGCCYGYRTCILRHVNPMRIHRAHDVCKHTDCYSPCE | | | | | | | | | | | | | | | |  |
| G56 | | yes | | | yes | | | 0 | 0 | | 70 | | | 77 | |  |
| MAMSMSMTLSVFVMVVMAATVTQLKKPDLSRMKRYDRRCCINKTYECLKNYRNRENKFASFCQQEAAVYCGTFESGTGCCYGYMNCMMINAERDGREKAHGYCQHRIC | | | | | | | | | | | | | | | |  |
| G57 | | yes | | | yes | | | 0 | 0 | | 30 | | | 72 | |  |
| MAMSMSMTLSVFVMVVMAATVTQLKKPDLSRMKRYDRRCCINKTYECLKNYRNRENKFASFCQQEAAVYCGTFESGTGCCYGYMNCMMLNAERDGQGKAHGYCQHRTC | | | | | | | | | | | | | | | |  |
| G58 | | | yes | | | yes | | | 0 | 0 | | 136 | | | 391 | |
| MAMSMSMTLSVFVMVVMAATVTGFTHLQVPNLSRMERDEPLDCCEMKKCCVRSMYECLQDHRGNEYDMATECYQKAGDICGSYNEIVGCCQGYEICILKNVPHTGLQRAHDLCESTDCYNPCQ | | | | | | | | | | | | | | | |  |
| G59 | | | yes | | | yes | | | 2 | 9 | | 2348 | | | 1339 | |
| MAMNMSMTLSVFVMVVMAATVTGFTQLKKPDLSRMKRDNKACCTNAIYQCLKRNPGQESYNTPPCHHAATTRCPGSHIDGCCPKYATCMSVNAQNNLETAHTYCLPRPCFDPCE | | | | | | | | | | | | | | | |  |
| **Conkunitzin** | | | | | | | | | | | | | | | |  |
| G60 | no | | | yes | | | 6 | | 4 | | 2 | | 0 | | |  |
| MEGRRFAAVLILTICMLAPGAVVSRRRVQPSECKLPAAKGPCKGKYRKVYFNNFKKQCRMFTYGGCGGNGNKFRNAKECYHKCAYGVG | | | | | | | | | | | | | | | |  |
| **Z-superfamily** | | | | | | | | | | | | | | | |  |
| G61 | no | | | yes | | | 0 | | 1 | | 0 | | 0 | | |  |
| MKLSVMFIVFLMLTMPMTCAGISRRADNGGEAGALSGDRAANIMALLQARGCPPMCNPGCHNCS | | | | | | | | | | | | | | | |  |
| **Contulakin G** | | | | | | | | | | | | | | | |  |
| G62  ([11]) | yes | | | no | | | 0 | | 0 | | 215 | | 1016 | | |  |
| MQTAYWVMVMMMVWIAAPLSEGGKLNDVIRGLVPDDITPQLILGSLISRRQSEEGGSNATKKPYILRASDQVASGP | | | | | | | | | | | | | | | |  |
| **Conophysin** | | | | | | | | | | | | | | | |  |
| G63 | yes | | | yes | | | 5 | | 12 | | 9 | | 228 | | |  |
| MTRSAMQMGRLTLVLCLLLLLLLTTQACFITNCPVGGKRHVEATHPCMSCSFGQCVGPQICCGLGGCEMGTAEANKCIEEDDDQTPCQVLGDHCDLNNLDIEGHCVADGICCVDDTCAIHSSC | | | | | | | | | | | | | | | |  |
|  | | | | | | | | | | | | | | | |  |

1. Nicke A, Loughnan ML, Millard EL, Alewood PF, Adams DJ, Daly NL, Craik DJ, Lewis RJ: **Isolation, structure, and activity of GID, a novel alpha 4/7-conotoxin with an extended N-terminal sequence**. *J Biol Chem* 2003, **278**(5):3137-3144.

2. Gray WR, Luque A, Olivera BM, Barrett J, Cruz LJ: **Peptide toxins from Conus geographus venom**. *J Biol Chem* 1981, **256**(10):4734-4740.

3. McIntosh JM, Olivera BM, Cruz LJ, Gray WR: **Gamma-carboxyglutamate in a neuroactive toxin**. *J Biol Chem* 1984, **259**(23):14343-14346.

4. Cruz LJ, Gray WR, Olivera BM, Zeikus RD, Kerr L, Yoshikami D, Moczydlowski E: **Conus geographus toxins that discriminate between neuronal and muscle sodium channels**. *J Biol Chem* 1985, **260**(16):9280-9288.

5. Olivera BM, McIntosh JM, Cruz LJ, Luque FA, Gray WR: **Purification and sequence of a presynaptic peptide toxin from Conus geographus venom**. *Biochemistry* 1984, **23**(22):5087-5090.

6. Yanagawa Y, Abe T, Satake M, Odani S, Suzuki J, Ishikawa K: **A novel sodium channel inhibitor from Conus geographus: purification, structure, and pharmacological properties**. *Biochemistry* 1988, **27**(17):6256-6262.

7. Olivera BM, Gray WR, Zeikus R, McIntosh JM, Varga J, Rivier J, de Santos V, Cruz LJ: **Peptide neurotoxins from fish-hunting cone snails**. *Science* 1985, **230**(4732):1338-1343.

8. Walker C. SR, Olivera B.M., Hooper D., Jacobsen R., Steele D., Jones R.M.: **US6630573**. In*.*; 2003.

9. England LJ, Imperial J, Jacobsen R, Craig AG, Gulyas J, Akhtar M, Rivier J, Julius D, Olivera BM: **Inactivation of a serotonin-gated ion channel by a polypeptide toxin from marine snails**. *Science* 1998, **281**(5376):575-578.

10. Olivera BM, Rivier J, Clark C, Ramilo CA, Corpuz GP, Abogadie FC, Mena EE, Woodward SR, Hillyard DR, Cruz LJ: **Diversity of Conus neuropeptides**. *Science* 1990, **249**(4966):257-263.

11. Craig AG, Norberg T, Griffin D, Hoeger C, Akhtar M, Schmidt K, Low W, Dykert J, Richelson E, Navarro V *et al*: **Contulakin-G, an O-glycosylated invertebrate neurotensin**. *J Biol Chem* 1999, **274**(20):13752-13759.
