# Supplementary material for: Elucidation of the molecular envenomation strategy of the cone snail Conus geographus through transcriptome sequencing of its venom duct
Source: BMC Genomics. 2012 Jun 28;13:284. doi: 10.1186/1471-2164-13-284 (PMC3441800; doi:10.1186/1471-2164-13-284)
Supplement: Additional file 2: Table S1. — A list of complete conotoxins sequences identified in the venom duct. The expression levels are shown for each conotoxin in each segment, represented as number of reads aligned to the toxin. Toxins are numbered G1-63, and have been listed according to their superfamilies. In the A superfamily, (X,Y) refers to the number of amino acid residues in the first and second disulfide loops. G4.x is the commonly used nomenclature of alpha-A family of conotoxins identified from Conus geographus. Other designations in parenthesis adjacent to GX indicate previously used nomenclature in the literature [3,4,12,49-55]. [file 1471-2164-13-284-S2.doc]

**Supplemental table 2.** InterPro protein families differentially expressed among the four segments Proximal (P), Proximal Central (PC), Distal Central (DC) and Distal (D), showing the proportion of aligned reads among whole transcriptome.

| IPR ID | Description | p-value | P | PC | DC | D |
| --- | --- | --- | --- | --- | --- | --- |
| IPR016179 | Insulin-like | 0.0E+00 | 0.E+00 | 1.5E-06 | 5.3E-06 | 1.6E-03 |
| IPR004161 | Translation elongation factor EFTu/EF1A, domain 2 | 0.0E+00 | 7.6E-04 | 1.3E-03 | 7.5E-04 | 5.1E-04 |
| IPR008331 | Ferritin/Dps protein | 0.0E+00 | 6.1E-04 | 2.2E-03 | 7.1E-04 | 4.8E-04 |
| IPR002223 | Proteinase inhibitor I2, Kunitz metazoa | 0.0E+00 | 1.8E-03 | 1.6E-03 | 4.9E-04 | 2.4E-04 |
| IPR000298 | Cytochrome c oxidase, subunit III | 0.0E+00 | 3.4E-04 | 5.6E-04 | 1.9E-04 | 1.9E-04 |
| IPR005822 | Ribosomal protein L13 | 0.0E+00 | 6.6E-05 | 2.8E-04 | 1.3E-04 | 1.7E-04 |
| IPR001506 | Peptidase M12A, astacin | 0.0E+00 | 8.5E-06 | 1.5E-06 | 7.5E-06 | 1.2E-04 |
| IPR001377 | Ribosomal protein S6e | 0.0E+00 | 5.4E-05 | 3.2E-04 | 8.1E-05 | 1.1E-04 |
| IPR022309 | Ribosomal protein S8e/ribosomal biogenesis NSA2 | 0.0E+00 | 1.1E-04 | 2.7E-04 | 7.2E-05 | 8.9E-05 |
| IPR011497 | Protease inhibitor, Kazal-type | 0.0E+00 | 1.9E-04 | 4.5E-04 | 9.2E-05 | 7.8E-05 |
| IPR000554 | Ribosomal protein S7e | 0.0E+00 | 8.8E-05 | 2.8E-04 | 6.0E-05 | 7.2E-05 |
| IPR005324 | Ribosomal protein S5, C-terminal | 0.0E+00 | 5.6E-05 | 2.4E-04 | 7.2E-05 | 7.1E-05 |
| IPR009779 | Translocon-associated, gamma subunit | 0.0E+00 | 1.4E-05 | 2.6E-05 | 1.9E-05 | 5.9E-05 |
| IPR002942 | RNA-binding S4 | 0.0E+00 | 1.1E-04 | 2.9E-04 | 7.4E-05 | 5.8E-05 |
| IPR001063 | Ribosomal protein L22/L17 | 0.0E+00 | 3.4E-05 | 2.1E-04 | 5.3E-05 | 5.7E-05 |
| IPR001865 | Ribosomal protein S2 | 0.0E+00 | 7.3E-05 | 2.2E-04 | 6.4E-05 | 5.6E-05 |
| IPR016082 | Ribosomal protein L30, ferredoxin-like fold domain | 0.0E+00 | 5.8E-05 | 2.0E-04 | 6.3E-05 | 5.3E-05 |
| IPR000266 | Ribosomal protein S17 | 0.0E+00 | 3.9E-05 | 1.8E-04 | 4.7E-05 | 5.3E-05 |
| IPR013843 | Ribosomal protein S4e, N-terminal | 0.0E+00 | 4.8E-05 | 2.1E-04 | 5.8E-05 | 4.9E-05 |
| IPR020040 | Ribosomal protein L6, alpha-beta domain | 0.0E+00 | 3.7E-05 | 1.8E-04 | 2.6E-05 | 4.5E-05 |
| IPR018940 | Elongation factor 1 beta central acidic region, eukaryote | 0.0E+00 | 5.9E-05 | 1.8E-04 | 3.6E-05 | 4.0E-05 |
| IPR001380 | Ribosomal protein L13e | 0.0E+00 | 4.8E-05 | 1.8E-04 | 3.5E-05 | 3.7E-05 |
| IPR022666 | Ribosomal Proteins L2, RNA binding domain | 0.0E+00 | 2.4E-05 | 1.6E-04 | 4.3E-05 | 3.7E-05 |
| IPR005326 | Plectin/S10, N-terminal | 0.0E+00 | 2.7E-05 | 2.1E-04 | 3.7E-05 | 3.0E-05 |
| IPR021131 | Ribosomal protein L18e/L15 | 0.0E+00 | 3.2E-05 | 1.5E-04 | 3.3E-05 | 3.0E-05 |
| IPR000885 | Fibrillar collagen, C-terminal | 0.0E+00 | 2.3E-04 | 2.9E-05 | 1.5E-05 | 2.5E-05 |
| IPR000209 | Peptidase S8/S53, subtilisin/kexin/sedolisin | 0.0E+00 | 1.9E-04 | 4.4E-04 | 8.8E-05 | 1.4E-05 |
| IPR001148 | Carbonic anhydrase, alpha-class, catalytic domain | 0.0E+00 | 0.0E+00 | 0.0E+00 | 2.2E-04 | 5.8E-06 |
| IPR001210 | Ribosomal protein S17e | 0.0E+00 | 1.8E-04 | 2.3E-04 | 6.2E-05 | 4.1E-06 |
| IPR018155 | Hyaluronidase | 1.1E-16 | 1.3E-04 | 5.3E-05 | 1.8E-05 | 9.1E-06 |
| IPR004000 | Actin-like | 2.7E-15 | 2.4E-04 | 2.9E-04 | 2.4E-04 | 2.7E-04 |
| IPR004044 | K Homology, type 2 | 3.4E-15 | 5.1E-05 | 1.5E-04 | 4.6E-05 | 2.8E-05 |
| IPR019636 | Cell wall-associated hydrolase | 4.9E-15 | 1.2E-03 | 7.7E-04 | 9.5E-04 | 6.2E-04 |
| IPR000235 | Ribosomal protein S7 | 5.3E-15 | 3.4E-05 | 1.5E-04 | 5.3E-05 | 4.0E-05 |
| IPR004099 | Pyridine nucleotide-disulphide oxidoreductase, dimerisation | 1.2E-14 | 1.7E-06 | 0.0E+00 | 8.6E-06 | 2.8E-05 |
| IPR000568 | ATPase, F0 complex, subunit A | 1.3E-14 | 6.8E-05 | 2.0E-04 | 6.7E-05 | 9.2E-05 |
| IPR000504 | RNA recognition motif domain | 1.6E-14 | 2.7E-04 | 5.0E-04 | 2.7E-04 | 2.4E-04 |
| IPR011759 | Cytochrome C oxidase subunit II, transmembrane domain | 2.8E-14 | 8.2E-05 | 2.3E-04 | 1.0E-04 | 1.2E-04 |
| IPR002429 | Cytochrome c oxidase subunit II C-terminal | 1.3E-13 | 3.2E-04 | 4.2E-04 | 2.1E-04 | 2.8E-04 |
| IPR002715 | Nascent polypeptide-associated complex NAC | 3.0E-13 | 1.0E-04 | 2.5E-04 | 1.0E-04 | 1.0E-04 |
| IPR005484 | Ribosomal protein L18/L5 | 7.5E-13 | 6.8E-05 | 1.9E-04 | 7.0E-05 | 6.2E-05 |
| IPR004038 | Ribosomal protein L7Ae/L30e/S12e/Gadd45 | 9.7E-13 | 9.2E-05 | 2.2E-04 | 8.0E-05 | 7.3E-05 |
| IPR001971 | Ribosomal protein S11 | 1.1E-12 | 3.6E-05 | 1.2E-04 | 2.8E-05 | 4.0E-05 |
| IPR016180 | Ribosomal protein L10e/L16 | 1.5E-12 | 2.5E-05 | 1.2E-04 | 4.1E-05 | 2.4E-05 |
| IPR002132 | Ribosomal protein L5 | 3.9E-12 | 1.4E-05 | 8.4E-05 | 2.5E-05 | 1.3E-05 |
| IPR000477 | Reverse transcriptase | 1.2E-11 | 1.3E-04 | 2.1E-04 | 1.2E-04 | 4.0E-05 |
| IPR000196 | Ribosomal protein L19/L19e | 1.5E-11 | 2.4E-05 | 1.1E-04 | 3.5E-05 | 2.3E-05 |
| IPR002136 | Ribosomal protein L4/L1e | 1.9E-11 | 3.4E-05 | 1.1E-04 | 2.6E-05 | 2.9E-05 |
| IPR000592 | Ribosomal protein S27e | 2.4E-11 | 1.3E-04 | 2.7E-04 | 1.2E-04 | 1.0E-04 |
| IPR000449 | Ubiquitin-associated/translation elongation factor EF1B, N-terminal | 2.9E-11 | 4.9E-05 | 1.6E-04 | 6.0E-05 | 5.0E-05 |
| IPR001564 | Nucleoside diphosphate kinase | 1.3E-10 | 9.9E-05 | 2.0E-04 | 6.7E-05 | 6.3E-05 |
| IPR001593 | Ribosomal protein S3Ae | 1.3E-10 | 8.0E-05 | 1.8E-04 | 7.0E-05 | 5.5E-05 |
| IPR022628 | S-adenosylmethionine synthetase, N-terminal | 2.6E-10 | 2.1E-04 | 2.6E-04 | 1.1E-04 | 6.3E-05 |
| IPR000597 | Ribosomal protein L3 | 4.1E-10 | 3.4E-05 | 1.2E-04 | 3.8E-05 | 3.0E-05 |
| IPR021138 | Ribosomal protein L18ae/LX | 6.4E-10 | 9.2E-05 | 1.8E-04 | 9.7E-05 | 1.2E-04 |
| IPR001372 | Dynein light chain, type 1/2 | 8.0E-10 | 3.1E-05 | 1.2E-04 | 5.0E-05 | 5.6E-05 |
| IPR005798 | Cytochrome b/b6, C-terminal | 9.3E-10 | 1.9E-04 | 3.4E-04 | 1.9E-04 | 1.8E-04 |
| IPR001848 | Ribosomal protein S10 | 1.2E-09 | 2.7E-05 | 9.5E-05 | 2.6E-05 | 2.2E-05 |
| IPR000439 | Ribosomal protein L15e | 2.3E-09 | 1.7E-05 | 9.5E-05 | 3.3E-05 | 3.9E-05 |
| IPR000754 | Ribosomal protein S9 | 3.0E-09 | 1.5E-05 | 6.6E-05 | 1.2E-05 | 1.4E-05 |
| IPR019781 | WD40 repeat, subgroup | 4.2E-09 | 1.3E-04 | 2.6E-04 | 1.4E-04 | 1.1E-04 |
| IPR014731 | Electron transfer flavoprotein, alpha subunit, C-terminal | 3.3E-08 | 5.1E-06 | 3.0E-05 | 3.2E-06 | 8.2E-07 |
| IPR010487 | Neugrin-related | 8.7E-08 | 0.0E+00 | 0.0E+00 | 4.3E-06 | 1.4E-05 |
| IPR018105 | Translationally controlled tumour protein | 2.0E-07 | 4.4E-04 | 5.7E-04 | 3.1E-04 | 2.5E-04 |
| IPR001266 | Ribosomal protein S19e | 3.6E-07 | 2.4E-05 | 7.4E-05 | 1.6E-05 | 2.8E-05 |
| IPR008160 | Collagen triple helix repeat | 4.4E-07 | 5.3E-05 | 6.0E-06 | 1.3E-05 | 6.6E-06 |
| IPR013025 | Ribosomal protein L25/L23 | 4.9E-07 | 2.2E-05 | 6.6E-05 | 1.4E-05 | 1.6E-05 |
| IPR006121 | Heavy metal transport/detoxification protein | 6.0E-07 | 1.9E-04 | 1.7E-04 | 8.7E-05 | 4.5E-05 |
| IPR002143 | Ribosomal protein L1 | 1.1E-06 | 1.9E-05 | 8.0E-05 | 3.1E-05 | 2.7E-05 |
| IPR001457 | NADH:ubiquinone/plastoquinone oxidoreductase, chain 6 | 5.6E-06 | 4.6E-05 | 9.8E-05 | 3.0E-05 | 4.4E-05 |
| IPR011701 | Major facilitator superfamily MFS-1 | 1.6E-05 | 3.7E-05 | 5.0E-05 | 1.4E-05 | 4.9E-06 |
| IPR001952 | Alkaline phosphatase | 2.2E-05 | 0.0E+00 | 0.0E+00 | 1.1E-06 | 8.2E-06 |
| IPR001854 | Ribosomal protein L29 | 3.1E-05 | 2.9E-05 | 5.6E-05 | 2.2E-05 | 6.6E-06 |

1. Nicke A, Loughnan ML, Millard EL, Alewood PF, Adams DJ, Daly NL, Craik DJ, Lewis RJ: **Isolation, structure, and activity of GID, a novel alpha 4/7-conotoxin with an extended N-terminal sequence**. *J Biol Chem* 2003, **278**(5):3137-3144.

2. Gray WR, Luque A, Olivera BM, Barrett J, Cruz LJ: **Peptide toxins from Conus geographus venom**. *J Biol Chem* 1981, **256**(10):4734-4740.

3. McIntosh JM, Olivera BM, Cruz LJ, Gray WR: **Gamma-carboxyglutamate in a neuroactive toxin**. *J Biol Chem* 1984, **259**(23):14343-14346.

4. Cruz LJ, Gray WR, Olivera BM, Zeikus RD, Kerr L, Yoshikami D, Moczydlowski E: **Conus geographus toxins that discriminate between neuronal and muscle sodium channels**. *J Biol Chem* 1985, **260**(16):9280-9288.

5. Olivera BM, McIntosh JM, Cruz LJ, Luque FA, Gray WR: **Purification and sequence of a presynaptic peptide toxin from Conus geographus venom**. *Biochemistry* 1984, **23**(22):5087-5090.

6. Yanagawa Y, Abe T, Satake M, Odani S, Suzuki J, Ishikawa K: **A novel sodium channel inhibitor from Conus geographus: purification, structure, and pharmacological properties**. *Biochemistry* 1988, **27**(17):6256-6262.

7. Olivera BM, Gray WR, Zeikus R, McIntosh JM, Varga J, Rivier J, de Santos V, Cruz LJ: **Peptide neurotoxins from fish-hunting cone snails**. *Science* 1985, **230**(4732):1338-1343.

8. Walker C. SR, Olivera B.M., Hooper D., Jacobsen R., Steele D., Jones R.M.: **US6630573**. In*.*; 2003.

9. England LJ, Imperial J, Jacobsen R, Craig AG, Gulyas J, Akhtar M, Rivier J, Julius D, Olivera BM: **Inactivation of a serotonin-gated ion channel by a polypeptide toxin from marine snails**. *Science* 1998, **281**(5376):575-578.

10. Olivera BM, Rivier J, Clark C, Ramilo CA, Corpuz GP, Abogadie FC, Mena EE, Woodward SR, Hillyard DR, Cruz LJ: **Diversity of Conus neuropeptides**. *Science* 1990, **249**(4966):257-263.

11. Craig AG, Norberg T, Griffin D, Hoeger C, Akhtar M, Schmidt K, Low W, Dykert J, Richelson E, Navarro V *et al*: **Contulakin-G, an O-glycosylated invertebrate neurotensin**. *J Biol Chem* 1999, **274**(20):13752-13759.
