# Supplementary material for: Elucidation of the molecular envenomation strategy of the cone snail Conus geographus through transcriptome sequencing of its venom duct
Source: BMC Genomics. 2012 Jun 28;13:284. doi: 10.1186/1471-2164-13-284 (PMC3441800; doi:10.1186/1471-2164-13-284)
Supplement: Additional file 3: Table S2. — InterPro protein families differentially expressed among the four segments Proximal (P), Proximal Central (PC), Distal Central (DC) and Distal (D), showing the proportion of aligned reads among whole transcriptome [[3],[4],[12],[49-55]]. [file 1471-2164-13-284-S3.doc]

**Supplemental Table 3.** Comparison of toxin sequences of αA-OIVA, a fetal muscle nicotinic acetylcholine receptor antagonist, with G10 4.1 and G11 4.2.

| αA-OIVA | **CC**GVONAA**C**HO**C**V**C**KNT**C**# |
| --- | --- |
| G10 4.1 | **CC**GKPNAA**C**HP**C**V**C**NGS**C**S# |
| G11 4.2 | **CC**GKPNAA**C**HP**C**V**C**NGS**C**SG# |

1. Nicke A, Loughnan ML, Millard EL, Alewood PF, Adams DJ, Daly NL, Craik DJ, Lewis RJ: **Isolation, structure, and activity of GID, a novel alpha 4/7-conotoxin with an extended N-terminal sequence**. *J Biol Chem* 2003, **278**(5):3137-3144.

2. Gray WR, Luque A, Olivera BM, Barrett J, Cruz LJ: **Peptide toxins from Conus geographus venom**. *J Biol Chem* 1981, **256**(10):4734-4740.

3. McIntosh JM, Olivera BM, Cruz LJ, Gray WR: **Gamma-carboxyglutamate in a neuroactive toxin**. *J Biol Chem* 1984, **259**(23):14343-14346.

4. Cruz LJ, Gray WR, Olivera BM, Zeikus RD, Kerr L, Yoshikami D, Moczydlowski E: **Conus geographus toxins that discriminate between neuronal and muscle sodium channels**. *J Biol Chem* 1985, **260**(16):9280-9288.

5. Olivera BM, McIntosh JM, Cruz LJ, Luque FA, Gray WR: **Purification and sequence of a presynaptic peptide toxin from Conus geographus venom**. *Biochemistry* 1984, **23**(22):5087-5090.

6. Yanagawa Y, Abe T, Satake M, Odani S, Suzuki J, Ishikawa K: **A novel sodium channel inhibitor from Conus geographus: purification, structure, and pharmacological properties**. *Biochemistry* 1988, **27**(17):6256-6262.

7. Olivera BM, Gray WR, Zeikus R, McIntosh JM, Varga J, Rivier J, de Santos V, Cruz LJ: **Peptide neurotoxins from fish-hunting cone snails**. *Science* 1985, **230**(4732):1338-1343.

8. Walker C. SR, Olivera B.M., Hooper D., Jacobsen R., Steele D., Jones R.M.: **US6630573**. In*.*; 2003.

9. England LJ, Imperial J, Jacobsen R, Craig AG, Gulyas J, Akhtar M, Rivier J, Julius D, Olivera BM: **Inactivation of a serotonin-gated ion channel by a polypeptide toxin from marine snails**. *Science* 1998, **281**(5376):575-578.

10. Olivera BM, Rivier J, Clark C, Ramilo CA, Corpuz GP, Abogadie FC, Mena EE, Woodward SR, Hillyard DR, Cruz LJ: **Diversity of Conus neuropeptides**. *Science* 1990, **249**(4966):257-263.

11. Craig AG, Norberg T, Griffin D, Hoeger C, Akhtar M, Schmidt K, Low W, Dykert J, Richelson E, Navarro V *et al*: **Contulakin-G, an O-glycosylated invertebrate neurotensin**. *J Biol Chem* 1999, **274**(20):13752-13759.
