# Supplementary material for: Elucidation of the molecular envenomation strategy of the cone snail Conus geographus through transcriptome sequencing of its venom duct
Source: BMC Genomics. 2012 Jun 28;13:284. doi: 10.1186/1471-2164-13-284 (PMC3441800; doi:10.1186/1471-2164-13-284)
Supplement: Additional file 4: Table S3. — Comparison of toxin sequences of αA-OIVA, a fetal muscle nicotinic acetylcholine receptor antagonist, with G10 4.1 and G11 4.2 [[3],[4],[12],[49-55]]. [file 1471-2164-13-284-S4.doc]

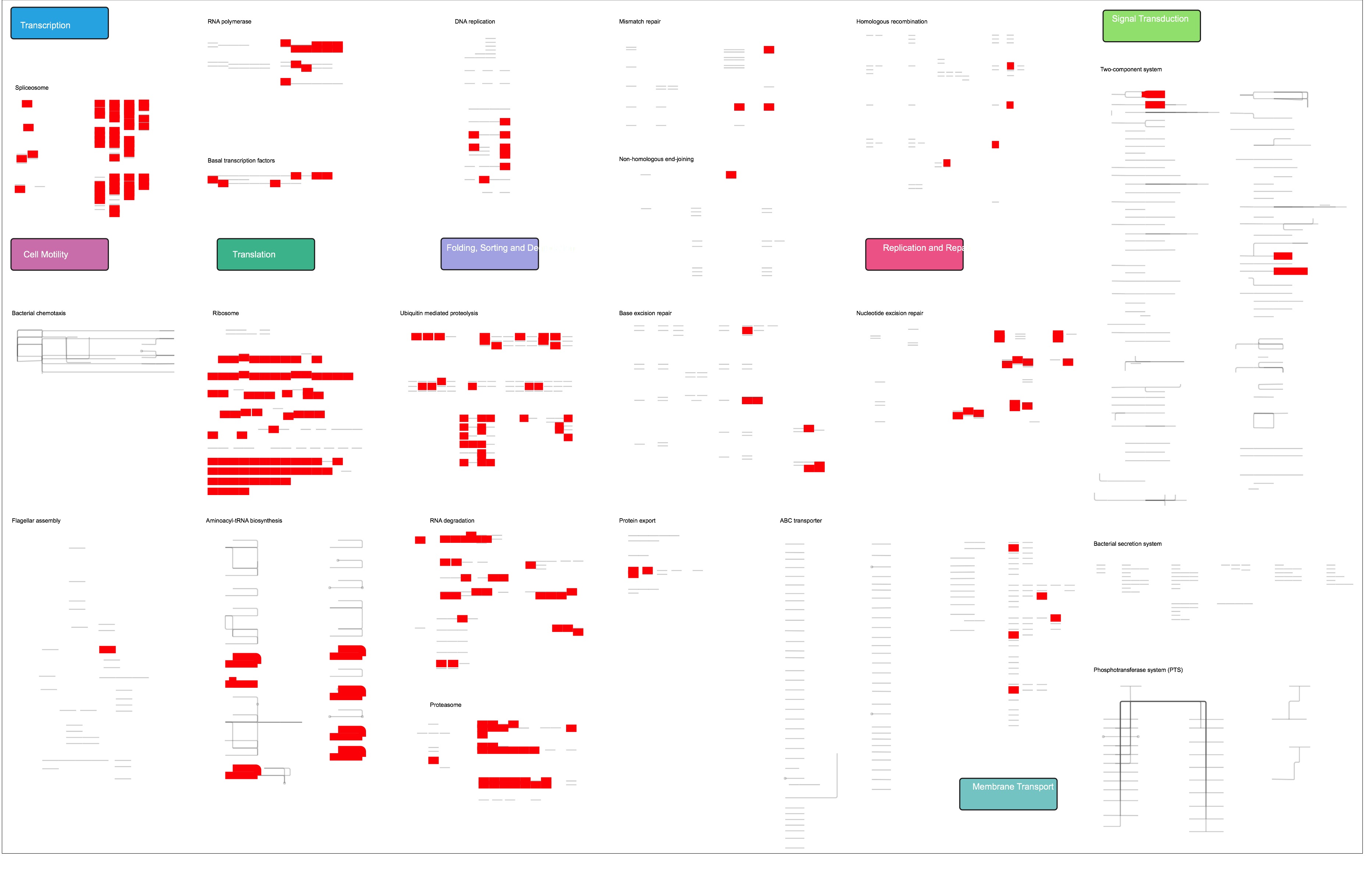


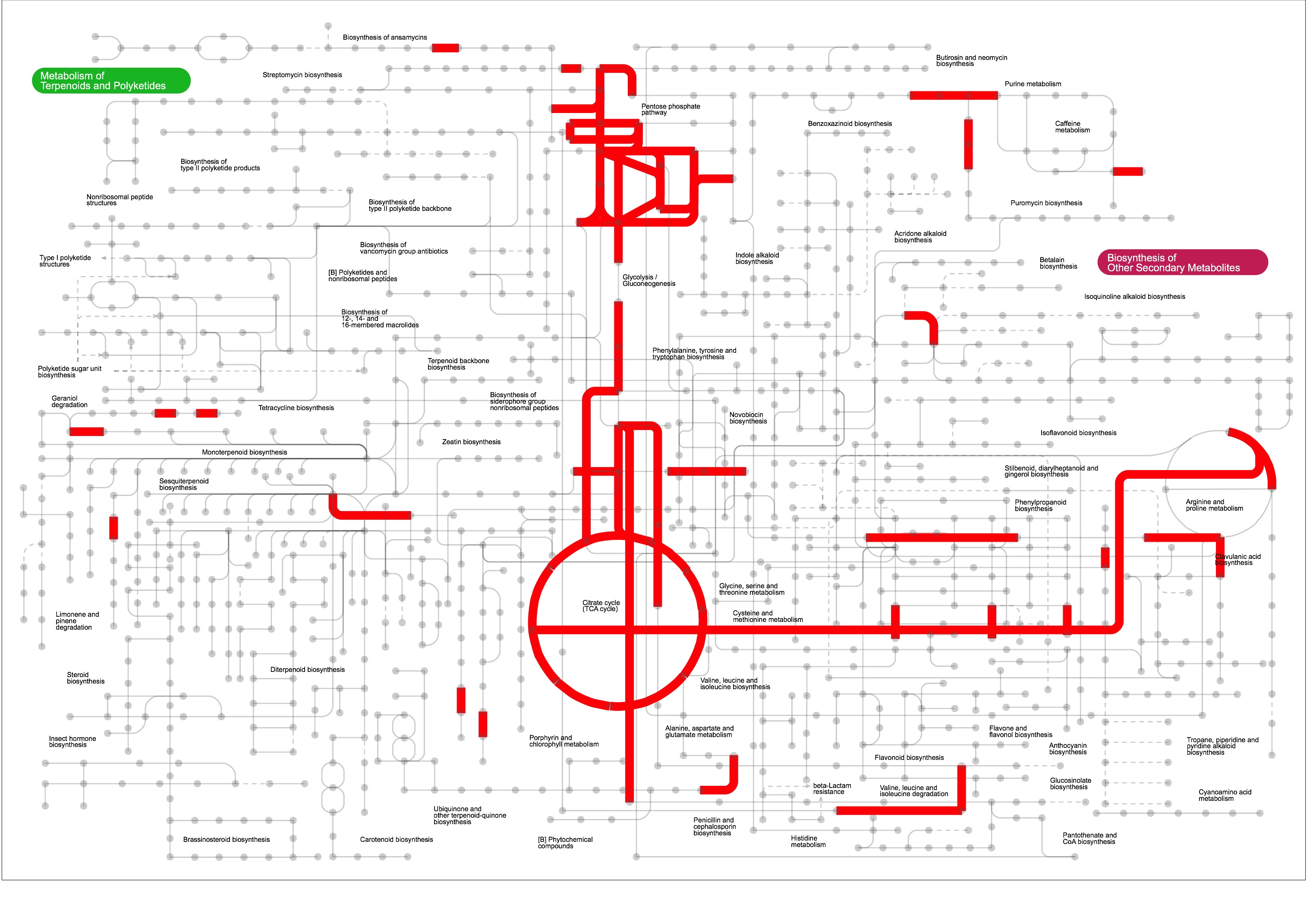


**Supplemental figure 1.** iPath (a) regulatory pathway map and (b) secondary-metabolite biosynthesis pathway map. Each grey dot represents a substrate and each red line represents an enzyme.
